# Left Atrial Dimension and CHA_2_DS_2_-VASc Score in Predicting Stroke in Patients with Rheumatic Atrial Fibrillation

**DOI:** 10.1016/j.jacadv.2024.101343

**Published:** 2024-10-30

**Authors:** Sheng-Nan Chang, Chih-Hsien Wang, Yoga Waranugraha, Ting-Tse Lin, Jien-Jiun Chen, Pang-Shuo Huang, Cho-Kai Wu, Yi-Chih Wang, Juey-Jen Hwang, Chia-Ti Tsai

**Affiliations:** a Division of Cardiology, Department of Internal Medicine, National Taiwan University Hospital Yun-Lin Branch, Yun-Lin, Taiwan; b Division of Cardiology, Department of Surgery, National Taiwan University Hospital, Taipei, Taiwan; c Department of Cardiology and Vascular Medicine, Brawijaya University Hospital, Malang, Indonesia; d Faculty of Medicine, Brawijaya University, Malang, Indonesia; e Division of Cardiology, Department of Internal Medicine, National Taiwan University Hospital, Taipei, Taiwan; f Graduate Institute of Clinical Medicine, College of Medicine, National Taiwan University, Taipei, Taiwan

**Keywords:** atrial fibrillation, CHA_2_DS_2_-VASc score, rheumatic heart disease, stroke

## Abstract

**Background:**

Rheumatic atrial fibrillation (AF) patients are at an elevated risk of stroke events, yet the associated risk factors remain unclear.

**Objectives:**

This study aimed to evaluate the effectiveness of the CHA_2_DS_2_-VASc score in predicting stroke events in rheumatic AF patients and explore potential enhancements for increased predicting accuracy in the Asian population, comparing it to nonvalvular AF.

**Methods:**

A retrospective cohort study spanning March 2010 to December 2020 included 29,341 AF patients followed up for up to 10 years, with 1,942 identified as having rheumatic AF. The CHA_2_DS_2_-VASc score was computed for all patients. Using the area under the receiver operating characteristic curve (AUC), we evaluated the performance of the predictive model. The clinical endpoint was ischemic stroke.

**Results:**

Rheumatic AF patients exhibited a higher stroke incidence than nonvalvular AF patients (log-rank *P* = 0.048). While the CHA_2_DS_2_-VASc score predicted stroke events in rheumatic AF, its AUC was lower than in nonvalvular AF (0.693 vs 0.746, *P* < 0.001). Integrating left atrial (LA) dimension information showed a trend toward an increased AUC for rheumatic AF to 0.707 (*P* = 0.068), but not for nonvalvular AF (AUC: 0.734 with LA, *P* = 0.744). The CHA_2_DS_2_-VASc-LA score showed a robust correlation with the annual incidence of ischemic stroke in rheumatic AF patients (*P* = 0.012 for trend).

**Conclusions:**

Rheumatic AF patients have a higher stroke risk than nonrheumatic AF and CHA_2_DS_2_-VASc-LA score might improve stroke risk assessment in this population.

Among elderly individuals, atrial fibrillation (AF) is the most common arrhythmia, and it is responsible for a significant proportion of strokes associated with aging.^1^ In geriatrics, AF associated with rheumatic heart disease (RHD) is rarely discussed. The leading causes of morbidity and mortality in this population are AF, heart failure (HF), and thromboembolism.^2^^,^^3^ AF affects around 32.8% of RHD patients, and the incidence nearly triples every 5 years following the first diagnosis.^4^ AF contributes to HF progression, systemic thromboembolism, and stroke.^4^, ^5^, ^6^ The characteristics of HF triggered by AF in RHD are "atrial kick" loss and fast irregular ventricular rates leading to the increased transmitral gradient. AF causes gradual left atrial (LA) enlargement over time, and many patients suffer from tachycardia-induced left ventricular (LV) systolic dysfunction as well. Several factors, including mitral valve involvement, LA dilatation, LA thrombus, LV systolic dysfunction, LA appendage contractility reduction, spontaneous echo contrast, and thromboembolism history, have been linked to increased stroke risk in rheumatic AF.^1^^,^^7^

AF patients have a greater risk of stroke and systemic thromboembolism,^8^ but that risk is not uniform and can be evaluated in individual patient settings using the CHA_2_DS_2_-VASc score.^9^ According to recent guidelines, administering oral anticoagulant (OAC) in nonvalvular AF has to be based on the CHA_2_DS_2_-VASc score. On the other hand, patients with valvular or rheumatic AF should receive vitamin K antagonist regardless of the CHA_2_DS_2_-VASc score.^10^^,^^11^ Those recommendations have been made because the CHA_2_DS_2_-VASc score has mainly been studied in populations of nonvalvular AF.^9^^,^^12^ For patients with the rheumatic AF subset, the predictive value of the CHA_2_DS_2_-VASc score still needs to be proven. Moreover, rheumatic AF patients have a greater risk of worsening LA dilatation, hypercoagulation, and possibly increasing stroke risk due to valvular abnormalities. Another essential issue is that Asian people are more sensitive and vulnerable to OAC.^13^ Therefore, we aimed to investigate the performance of the CHA_2_DS_2_-VASc score and, if possible, modify it to improve its performance in predicting stroke events in a large Asian population with rheumatic AF.

## Materials and methods

### Study design and study population

This was a retrospective longitudinal follow-up cohort study. This cohort was a subsidiary study of our National Taiwan University Atrial Fibrillation Registry.^14^^,^^15^ All patients with permanent, persistent or paroxysmal AF were enrolled in this registry. The diagnosis of AF was determined by taking the patient’s history, serial electrocardiogram (ECG), and/or ambulatory ECG monitoring. A total of 29,341 patients were recruited and were followed up from March 2010 to December 2020 for a maximum of 10 years, until the first occurrence of ischemic stroke or until December 2020. Among 29,341 AF patients in this cohort, 1942 patients had mitral stenosis (MS) and were defined as those with rheumatic AF according to the guidelines.^16^^,^^17^ The Research Ethics Committee of the National Taiwan University Hospital approved the study protocol (200911002R). This observation cohort study was performed adhering to the observational cohort guideline.

### Definition of rheumatic AF

Patients with rheumatic AF were defined as those with AF and characteristic echocardiographic findings of rheumatic MS. The transthoracic echocardiographic findings of MS were the pathognomonic changes of mitral valve calcification with commissural fusion resulting in a diastolic “doming” pattern with the leaflet tips pointing toward each other in mid-diastole. The mitral valve orifice area was also measured at the mitral leaflet tips with the 2-dimensional mode or pressure-half-time method. The definition of MS was mitral valve area of <2.0 cm^2^. LA size, LV size, and LV systolic function were also assessed. Coexisting mitral, tricuspid, and aortic regurgitation were also evaluated. Nonvalvular AF was defined as cases without rheumatic MS and prosthetic heart valve.^18^

### Clinical and outcome assessments

All the baseline characteristics were collected as previously described.^14^^,^^19^^,^^20^ The medical diagnoses of hypertension (blood pressure consistently above 140/90 mm Hg or treated with hypertension medication), diabetes mellites (fasting glucose ≥125 mg/dL and HbA1C ≥6.5% or under oral hypoglycemic agent and/or insulin usage), HF (admission for congestive HF) and vascular disease (peripheral arterial disease, coronary artery disease, and myocardial infarction) were according to current guidelines.^18^

Adverse thromboembolic endpoints during follow-up were defined as incident episodes of ischemic stroke. Ischemic stroke was defined as sudden-onset and focal or global neurological deficits that were not explained by other origins, with supporting evidence from imaging studies. Hemorrhagic stroke was excluded from the study because it was generally a complication of using anticoagulants and not related to AF per se.

### Calculation of CHA_2_DS2-VASc and CHA_2_DS_2_-VASc-LA score

The CHA_2_DS_2_-VASc score assigned 2 points for stroke/transient ischemic attack and age ≥75 years, and 1 point for age 65 to 74 years, hypertension, diabetes, HF, female sex, and vascular disease. The CHA_2_DS_2_-VASC-LA score added LA dimension, with 1 point for 45 to 55 mm and 2 points for >55 mm, refining risk stratification.

### Statistical analyses

Continuous variables were presented as means ± SD, and categorical variables as percentages. Data comparisons used chi-square or Student’s *t*-tests. Stroke event timing was analyzed with Kaplan–Meier curves and log-rank tests. Cox regression was used to adjust for CHA_2_DS_2_-VASc score which represents the summary information including HF, hypertension, age, diabetes, stroke, vascular disease and sex, and HRs and 95% CIs were calculated. Receiver operating characteristic analysis, with the area under the curve (AUC) and c-statistics, assessed risk predictor discrimination. Sensitivity and specificity were calculated across cutoff values. Statistical analysis was conducted using STATA 16.0 (STATA, Inc), with significance set at *P* < 0.05.

## Results

### Baseline characteristics

The baseline characteristics of all AF patients with or without rheumatic MS are summarized in Table 1. The patients with rheumatic AF were younger; fewer were men, and fewer had comorbidities such as hypertension, diabetes, vascular disease, and coronary artery disease compared to nonvalvular AF. The prevalence of congestive HF was more significant in the rheumatic AF group. The mean CHADS_2_-VASc score was also lower in those with MS than in those without MS.

**Table 1:** Baseline Characteristics of Patients With Rheumatic AF and Nonvalvular AF

	Rheumatic AF (n = 1,942)	Nonvalvular AF (n = 27,399)	*P* Value
Age (y)	67.9 ± 13.5	71.2 ± 13.2	<0.001
Male	795 (40.9)	15,872 (57.9)	<0.001
History of stroke	338 (17.4)	5,138 (18.8)	0.141
CHF	683 (35.2)	5,135 (18.7)	<0.001
Hypertension	698 (35.9)	12,830 (46.8)	<0.001
DM	302 (15.6)	5,994 (21.9)	<0.001
CAD	276 (14.2)	5,270 (19.2)	<0.001
PAOD	44 (2.3)	773 (2.8)	0.150
CHA_2_DS_2_-VASc	2.77 ± 1.81	2.90 ± 1.82	0.004
LA dimension, cm	5.05 ± 1.10	4.21 ± 0.88	<0.001
LVEF, mm	62.67 ± 11.54	63.63 ± 12.49	<0.001
LVIDs, mm	3.22 ± 0.79	3.14 ± 0.78	<0.001
LVIDd, mm	4.87 ± 0.78	4.80 ± 0.70	<0.001
Use of warfarin	119 (61.4)	5,191 (18.9)	<0.001
Use of NOAC	482 (24.8)	8,427 (30.8)	<0.001
Use of antiplatelet	635 (32.7)	10,278 (37.5)	<0.001

Values are mean ± SD or n (%).

AF = atrial fibrillation; CAD = coronary artery disease; CHF = congestive heart failure; DM = diabetes mellitus; LA = left atrial; LVEF = left ventricular ejection fraction; LVIDs = left ventricular internal diameter in systole; LVIDd = left ventricular internal diameter in diastole; NOAC = non-vitamin K antagonist oral anticoagulant; PAOD = peripheral arterial occlusive disease.

Cardiac remodeling was more prominent in individuals with rheumatic AF than in individuals with nonvalvular AF. They have a bigger LA diameter and a larger LV internal diameter in systole. Patients with nonvalvular AF, on the other hand, had a greater LV ejection fraction. Warfarin use was higher in patients with rheumatic AF than in patients with nonvalvular AF. However, the use of non-vitamin K antagonist oral anticoagulant and antiplatelet was higher in the nonvalvular AF group.

### Comparison of stroke risk between patients with rheumatic AF and those with nonvalvular AF

**Figure 1:**
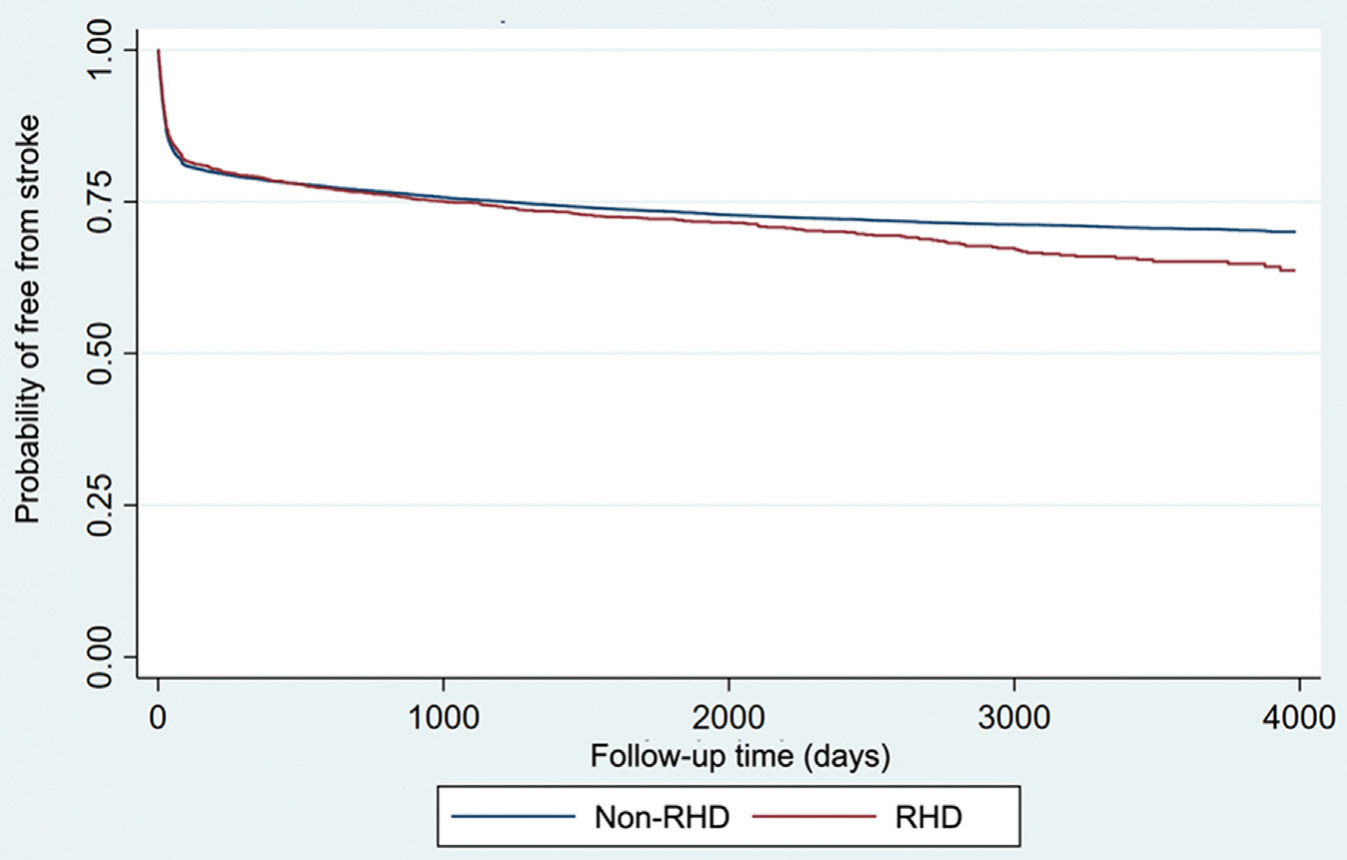
Stroke-Free Survival Comparison: Rheumatic AF vs Nonvalvular AF The event-free survival from stroke between rheumatic AF and non-valvular AF patients. AF = atrial fibrillation; RHD = rheumatic heart disease.

At the end of the follow-up, 7,075 nonvalvular AF patients had an incident event of ischemic stroke. The stroke rate was 6.15 per 100 patient-years (6.15%). At the end of the follow-up, 563 patients with rheumatic AF had an incident event of ischemic stroke. The stroke rate was 6.85 per 100 patient-years (6.85%) in those with rheumatic AF. Although patients with rheumatic AF had a lower mean CHADS_2_-VASc score, they had a higher stroke event rate than those with nonvalvular AF. The event-free survival from stroke between patients with rheumatic AF and those with nonvalvular AF is shown in Figure 1. Patients with rheumatic AF were more likely to have an incident stroke than those with nonvalvular AF (log-rank *P* = 0.048).

### CHA_2_DS_2_-VASc score as a predictor of incident stroke events in those with rheumatic AF

Because the CHA_2_DS_2_-VASC score is a well-established scoring scheme for patients with nonvalvular AF to predict the risk of stroke, we first tested whether the CHA_2_DS_2_-VASC score was associated with stroke in patients with nonvalvular AF in our cohort. As expected, the CHA_2_DS_2_-VASc score was incrementally related to the risk of stroke. Using the Cox model, we found a 55% increase in the risk of stroke with a 1-point increase in CHA_2_DS_2_-VASc score in this long-term follow-up cohort (HR: 1.55, 95% CI: 1.53-1.57, *P* < 0.001).

**Figure 2:**
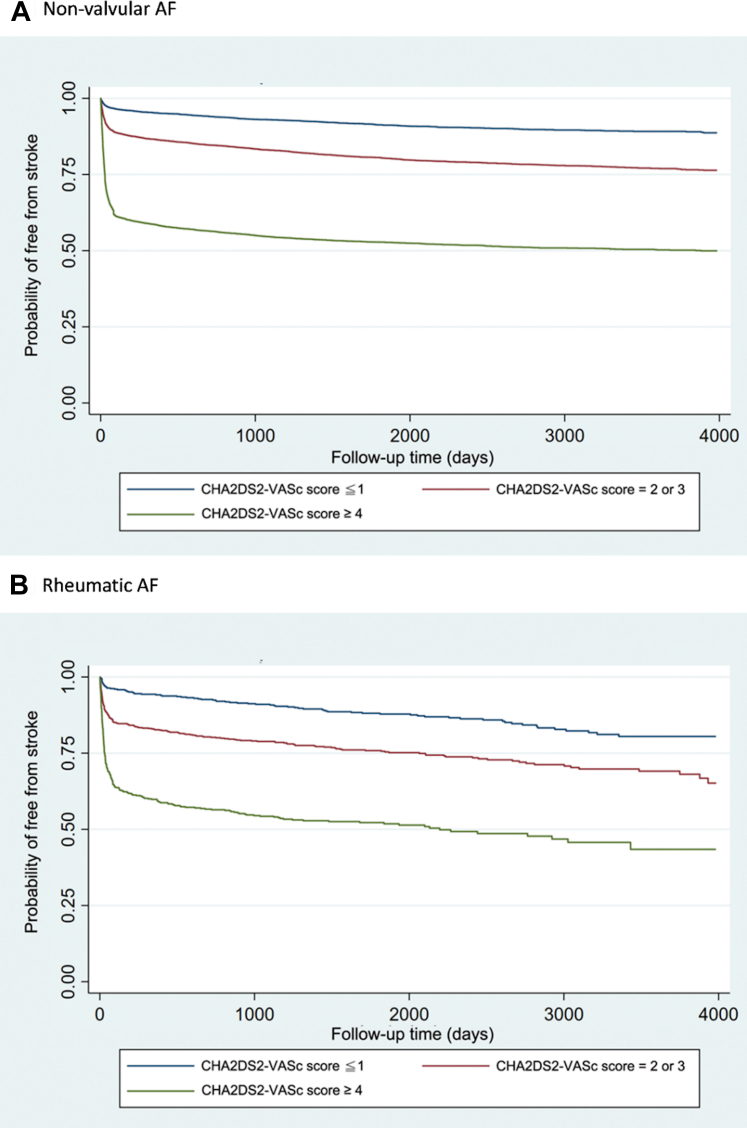
**Stroke-Free Survival by CHA_2_DS_2_-VASc Score: Nonvalvular vs Rheumatic AF** The event-free survival from stroke comparing patients with different CHA_2_DS_2_-VASC scores in non-valvular AF (A) and rheumatic AF (B). AF = atrial fibrillation.

We then evaluated whether the CHA_2_DS_2_-VASC score was also incrementally associated with the risk of stroke in those with rheumatic AF. We found a 44% increase in the risk of stroke with a 1-point rise in CHA_2_DS_2_-VASC score in this long-term follow-up cohort (HR: 1.44, 95% CI: 1.38-1.50, *P* < 0.001). The event-free survival from stroke comparing patients with different CHA_2_DS_2_-VASC scores is shown in Figure 2. In both patients with rheumatic AF and nonvalvular AF (Figure 2A and 2B), those with a higher CHA_2_DS_2_-VASC score were more likely to have an incident stroke than those with a lower CHA_2_DS_2_-VASC score (log-rank *P* < 0.001).

### Comparison of the performance of CHA_2_DS_2_-VASc score as a predictor of stroke between rheumatic AF and nonvalvular AF

**Figure 3:**
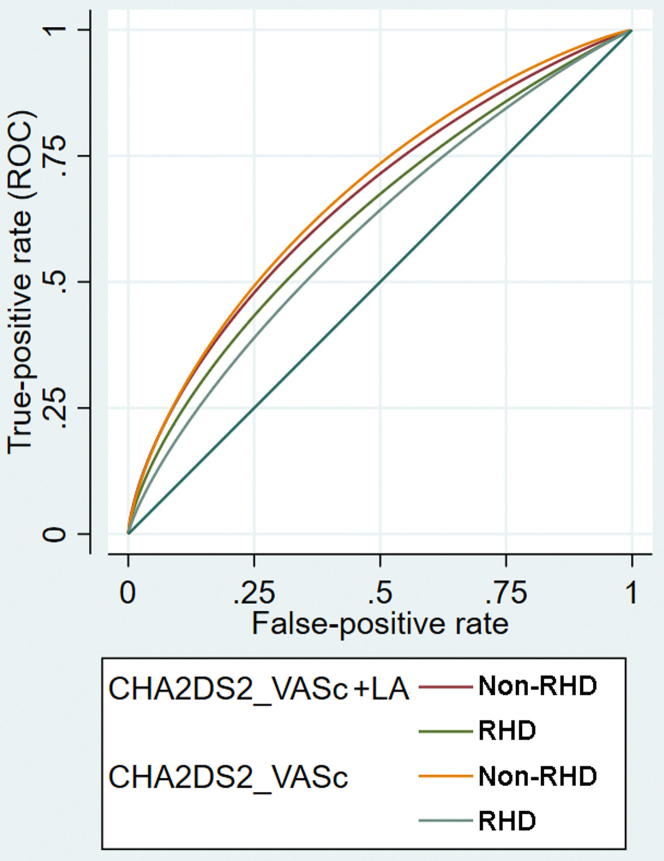
**ROC Analysis of CHA_2_DS_2_-VASc Score With or Without LA** ROC and c statistic to test the accuracy of the CHA_2_DS_2_-VASC score and CHA_2_DS_2_-VASC score plus LA information in predicting the occurrence of stroke in non-valvular AF and rheumatic AF. AF = atrial fibrillation; CHA_2_DS_2_-VASc = Congestive heart failure, Hypertension, Age ≥75 years, Diabetes mellitus, Stroke, Vascular disease, Age 65-74 years, Sex category (female); LA = left atrial; RHD = rheumatic heart disease; ROC = receiver operating characteristic.

Since the CHA_2_DS_2_-VASC score was associated with the risk of stroke in both patients with rheumatic AF and those with nonvalvular AF, we used receiver operating characteristic and *c* statistic (AUC statistic) to test the accuracy of the CHA_2_DS_2_-VASC score in predicting the occurrence of stroke (Figure 3). For the CHA_2_DS_2_-VASC score in those with rheumatic AF, the AUC or *c* statistic for stroke events was 0.693 (95% CI: 0.666-0.720). For those with nonvalvular AF, the AUC or *c* statistic for stroke events was 0.746 (95% CI: 0.736-0.756). The *c* statistic of the CHA_2_DS_2_-VASC score was significantly higher in those with nonvalvular AF than in those with rheumatic AF (*P* < 0.001).

### Association between LA dimension and stroke risk

We also assessed the association between LA dimension and stroke risk in both participant groups. In rheumatic AF patients, we found that greater LA dimension was associated with a greater risk of stroke (HR: 1.12, 95% CI: 1.02-1.23, *P* = 0.014). There was a 12% increase of stroke risk per 1 cm increase of LA dimension. However, in nonvalvular AF patients, we did not find an association between LA dimension and the risk of stroke (HR: 1.01, 95% CI: 0.97-1.04, *P* = 0.667).

### The addition of LA dimension in the CHA_2_DS_2_-VASC score to predict stroke

**Figure 4:**
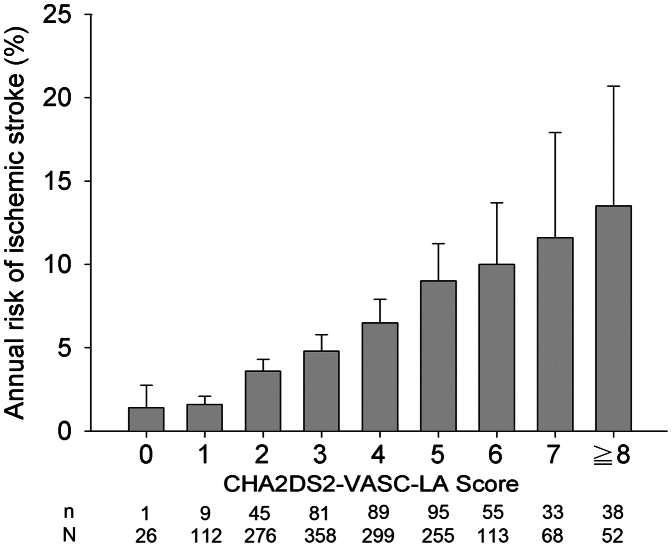
**CHA_2_DS_2_-VASc-LA Score and Annual Ischemic Stroke Risk** CHA_2_DS_2_-VASC-LA score and annual ischemic stroke risk. CHA_2_DS_2_-VASc = Congestive heart failure, Hypertension, Age ≥75 years, Diabetes mellitus, Stroke, Vascular disease, Age 65-74 years, Sex category (female), LA dimension; n = Patient number with stroke; N = total Patient number in each subgroup.

In patients with rheumatic AF, the AUC or c statistic showed a trend of increase when the information of the LA dimension was added to the CHA_2_DS_2_-VASC score (0.707 [95% CI: 0.678-0.737] vs 0.693 [95% CI: 0.666-0.720], *P* = 0.068) (Figure 3). However, in patients with nonvalvular AF, the AUC or c statistic did not increase (*P* = 0.342) when LA dimension data were added (AUC: 0.734, 95% CI: 0.724-0.744), compared to AUC with CHA_2_DS_2_-VASC score only (Figure 3). We proposed a CHA_2_DS_2_-VASC-LA score specifically for patients with rheumatic AF to determine the risk of stroke. One point was given for those with LA dimension >45 mm but <55 mm. Two points were given for those with an LA dimension of >55 mm. This CHA_2_DS_2_-VASC-LA score could predict the risk of stroke for patients with rheumatic AF with an AUC of 0.702 (95% CI: 0.668-0.740). This AUC was still numerically lower than that of patients with nonvalvular AF using CHA_2_DS_2_-VASC score (AUC: 0.702, 95% CI: 0.668-0.740 vs AUC: 0.746, 95% CI: 0.736-0.756, *P* = 0.102). Nevertheless, we found a strong relationship between the CHA_2_DS_2_-VASC-LA score and the annual incidence of ischemic stroke in the rheumatic AF population (*P* = 0.012 for trend). We found that when the CHA_2_DS_2_-VASC-LA score increased, the annual ischemic stroke risk also increased (Figure 4 and Central Illustration).

**Central Illustration:**
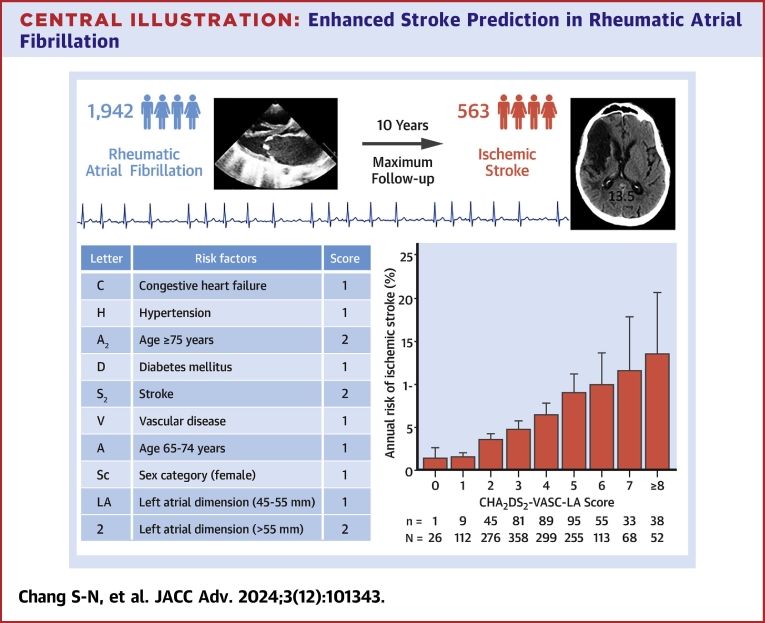
**Enhanced Stroke Prediction in Rheumatic Atrial Fibrillation** Rheumatic atrial fibrillation (AF) Patients face a higher Stroke risk. This study assessed the CHA_2_DS_2_-VASc score's predictive power for Stroke in rheumatic AF and explored the benefits of incorporating left atrial dimension. Results indicated that the CHA_2_DS_2_-VASc-LA score improved stroke risk prediction accuracy, highlighting its potential for better clinical assessment in this population.

A significant portions of AF patients were on OAC treatment. The predictive values of CHA_2_DS_2_-VASC score or LA dimension might by mitigated by concomitant use of OAC. Therefore, we did the analyses again separately in those with OAC and those without OAC. We found that in those with nonvalvular AF, the AUC of CHADS_2_-VASc score was significantly higher in those without OAC (0.780 [0.771-0.789] without OAC vs 0.719 [0.709-0.729] with OAC, *P* < 0.001). In those with rheumatic AF, the AUC of CHADS_2_-VASc score was numerically higher in those without OAC, but not statistically significant (0.732 [0.677-0.786] without OAC vs 0.709 [0.680-0.738] with OAC, *P* = 0.404). Furthermore, in patients with rheumatic AF, adding LA to CHADS_2_-VASc score resulted in a numerically higher AUC in those without OAC (AUC of CHADS_2_-VASc without LA 0.732 [0.677-0.786] and 0.749 [0.673-0.825] when LA added to CHADS_2_-VASc, *P* = 0.740), albeit not statistically significant, but not in those with OAC (0.709 [0.680-0.738] vs 0.711 [0.678–0.744], *P* = 0.155). In the patients with nonvalvular AF, adding LA to CHADS_2_-VASc score did not result in increased AUC, but decreased of AUC either in those with OAC (AUC of CHADS_2_-VASc 0.780 [0.771-0.789] vs CHADS_2_-VASc with LA 0.759 [0.744-0.774], *P* = 0.002) or those without OAC (AUC of CHADS_2_-VASc AUC 0.719 [0.709-0.729] vs CHADS_2_-VASc with LA 0.706 [0.693 0.719], *P* < 0.001).

## Discussion

There are several significant results to be highlighted from our study. First, in the Asian population, rheumatic AF patients were more likely than nonvalvular AF patients to suffer from stroke. Second, patients with rheumatic AF and a higher CHA_2_DS_2_-VASC score had a higher risk of incident stroke than those with a lower CHA_2_DS_2_-VASC score. Third, the performance of the CHA_2_DS_2_-VASC score was substantially better in nonvalvular AF patients than in rheumatic AF patients. Fourth, greater LA dimension was related to an increased risk of stroke in rheumatic AF patients. Finally, the CHA_2_DS_2_-VASC-LA score provided better performance in predicting stroke in rheumatic AF compared to the CHA_2_DS_2_-VASC score only.

RHD begins during childhood as acute rheumatic fever, caused by an autoimmune reaction to group A beta-hemolytic streptococcal (GAS) infection. In the beginning phase of the disease, the heart inflammation causes dilation of valve annuli rings, which surround the valve and aid seal leaflets during systole, as well as elongation of chordae tendinae, which connect mitral and tricuspid valve leaflets to the left and right ventricles, respectively. These alterations result in insufficient valve leaflet coaptation that causes regurgitation.^1^ Recurrent episode of acute rheumatic fever causes inflammation and fibrous tissue formation of the heart valves leading to RHD.^7^ Further inflammation causes fibrous tissue accumulation and scarring of the leaflets, which can eventually lead to valvular stenosis, a condition in which the valve becomes narrow, immobile, and cannot open fully.^1^ The first and recurrent damage and fibrosis pose a hemodynamic burden and cause further heart disease worsening in many patients. As time goes by, RHD causes not only congestive HF and pulmonary hypertension but also AF and thromboembolism.^7^ Complications in RHD patients rise with age, as does valvular dysfunction severity.^21^

AF is well known as the most common arrhythmia in RHD patients. Its prevalence is higher in patients with multiple and more complex valve diseases.^4^ Chronic LA pressure and volume overload are caused by left-sided valvular lesions, particularly mitral valve lesions, resulting in atrial inflammation and fibrosis. This inflammation process also plays a crucial role in myocardial disarray and fibrosis. A study that examined the pathology of excised LA appendage in RHD patients following mitral valve replacement discovered that LA tissue in AF exhibited severe degenerative remodeling and continuing inflammation with massive fibrosis.^22^ Compared to patients with sinus rhythm, the AF and mitral valve disease patients revealed more prominent atrial fibrosis and higher expression of advanced glycation end-product receptors.^23^ Electrically, those anatomical and functional changes promote effective refractory period variability and disrupt impulse propagation, resulting in the establishment of functional blocks. Increased atrial refractoriness spatial dispersion aggravates the nonuniform regional differences contributing to AF.^1^^,^^7^

Increased stroke risk in rheumatic AF has been linked to mitral valve disease, LA dilatation, LA thrombus, LV systolic dysfunction, LA appendage contractility decrease, spontaneous echo contrast, and prior thromboembolism.^1^^,^^7^ In this study, we found that during the long-term follow-up period, rheumatic AF patients were more likely than nonvalvular AF patients to suffer from a stroke than nonvalvular AF. However, data analysis from the RE-LY (Randomized Evaluation of the Long-Term Anticoagulation Therapy) AF registry revealed that the presence of the RHD did not significantly increase the stroke risk.^24^ The difference in results was due to differences in the follow-up duration, whereas the follow-up time in our study was much longer.

Our cohort initially examined whether the CHA_2_DS_2_-VASC score was linked with stroke in patients with nonvalvular AF because it is a well-established scoring method for patients with nonvalvular AF to predict the risk of stroke.^10^^,^^11^ The CHA_2_DS_2_-VASc score was, as expected, gradually related to the risk of stroke in this population subset. In those with rheumatic AF, we found that the CHA_2_DS_2_-VASC score was also incrementally associated with the risk of stroke. In this current study, the predictive value of the CHA_2_DS_2_-VASC score was substantially higher in nonvalvular AF patients than in rheumatic AF patients.

The performance of the CHA_2_DS_2_-VASc score in predicting stroke in nonvalvular AF was good (AUC = 0.746; 95% CI: 0.739-0.753). However, in rheumatic AF individuals, the performance of the CHA_2_DS_2_-VASc score was only “satisfactory” (AUC = 0.693; 95% CI: 0.666-0.720). Those results were slightly different from the prior study by Benz et al. that showed modest performance of the CHA_2_DS_2_-VASc score in AF patients with and without RHD.^24^

We recognized several reasons that could affect the results. First, in our study, the patient with rheumatic AF was predominated by younger patients. Second, the prevalence of hypertension, diabetes, and vascular disease, including coronary artery disease, was lower in the rheumatic AF group. Our previous cohort study found a favorable association between echocardiographic LA dimension and CHA_2_DS_2_-VASc score in the overall AF population. That study also demonstrated that LA dimension is not an independent predictor of AF-related stroke risk. Still, it does give a diagnostic value in predicting AF-related stroke risk due to its association with the CHA_2_DS_2_-VASc score.^25^ However, in our study, we also recognized that in the rheumatic AF population, the LA dimension was an independent predictor for stroke. The logical explanation for this phenomenon is that the autoimmune, inflammatory, and remodeling processes of the left atrium occur at a relatively younger age in the rheumatic AF population.^26^ Enlargement of the left atrium characterizes the remodeling process of the left atrium. Furthermore, individuals with rheumatic AF have minimal comorbidities that are components of the CHA_2_DS_2_-VASc score. Despite having a low CHA_2_DS_2_-VASc score, this population is at high risk of thromboembolism.

In rheumatic AF patients, combining the LA dimension with the original CHA_2_DS_2_-VASc score may provide a better performance in predicting stroke. In this study, we provided evidence regarding the better performance of the CHA_2_DS_2_-VASC-LA score for stroke risk prediction compared to the original CHA_2_DS_2_-VASC score. Our study revealed that when the CHA_2_DS_2_-VASC-LA score was more than or equal to 2, the risk of embolic stroke was at least 3.6%. The reported annual incidence of major hemorrhage, including hemorrhagic stroke, for warfarin users was 1 to 3%.^27^ Therefore, based on these results, we recommend that every patient with rheumatic AF and CHA_2_DS_2_-VASC-LA score of more than or equal to 2 needs warfarin therapy. The annual risk of stroke for patients with rheumatic AF and CHA_2_DS_2_-VASC-LA scores 0 and 1 was low (1.4% to 1.6%) (Figure 4), and they may not need warfarin therapy to take the 1 to 3% annual risk of hemorrhagic stroke.^27^

In the Kaplan-Meier curves presented in this study, a nonlinear pattern is observed, with a significant number of stroke events occurring shortly after enrollment, particularly in patients with high CHA_2_DS_2_-VASc scores (Figure 2). In studies with longer follow-up periods, higher-risk patients (eg, those with elevated CHA_2_DS_2_-VASc scores) may experience events earlier, leading to an initial dip in the Kaplan-Meier curve. As time progresses, and as higher-risk patients experience events while lower-risk patients remain event-free, the curve tends to flatten. This pattern suggests the possibility that the CHA_2_DS_2_-VASc score may be more effective in predicting early stroke events in our cohort. To assess whether a more linear risk prediction could be achieved, other risk scoring systems could be considered or developed.

### Study Limitations

First, this study included all patients including those with OAC and those without OAC for analyses. It may be more valid to do the analyses only in those without OAC, which might decrease the case number. Nevertheless, we also did subgroup analyses only in those without OAC. Second, we only used the first occurrence of ischemic stroke as the outcome, which might lead to underestimating the cardiovascular risk related to AF. Third, the c-statistic was used for model discrimination but did not assess calibration, misclassification errors, or risk prediction accuracy. Finally, data on the international normalized ratio and the time in therapeutic range for warfarin users were unavailable, and the study did not evaluate predictive performance across racial and ethnic groups, limiting its applicability primarily to Asian populations.

## Conclusions

In this cohort, including the Asian population, rheumatic AF patients were more likely than nonvalvular AF patients to suffer from stroke. The performance of the CHA_2_DS_2_-VASc score in predicting stroke in valvular AF was satisfactory. However, adding on the LA dimension information may be considered in assessing ischemic stroke risk in this population.

PERSPECTIVES **COMPETENCY IN MEDICAL KNOWLEDGE:** AF is common among the elderly and increases the risk of stroke, particularly in patients with RHD. Including LA dimension in the CHA2DS2-VASc score (CHA2DS2-VASc-LA score) may improve stroke risk prediction for patients with rheumatic AF.

**TRANSLATIONAL OUTLOOK:** This study underscores the necessity for multidisciplinary efforts to adapt and validate clinical tools for stroke risk prediction across diverse AF populations. By enhancing stroke risk stratification in patients with rheumatic AF, it bridges clinical practice, promotes biomedical research, and advances personalized medicine. Future research should evaluate the performance of the CHA2DS2-VASc-LA score in different ethnic populations.

## Funding support and author disclosures

This work was supported by grants from the National Science and Technology Council, Taiwan (grant numbers 113-2314-B-002-020- and 113-2314-B-002-147-MY3). The authors declare no financial or personal relationships with any individuals or organizations that could inappropriately influence or bias their work.
